# Nanomaterials Synthesis Discovery via Parallel Electrochemical Deposition

**DOI:** 10.1021/acs.chemmater.4c00318

**Published:** 2024-03-14

**Authors:** Michelle L. Personick, Abdoulie A. Jallow, Gabriel C. Halford, Lane A. Baker

**Affiliations:** † Department of Chemistry, University of Virginia, Charlottesville, Virginia 22904, United States; ‡ Department of Chemistry, Wesleyan University, Middletown, Connecticut 06459, United States; § Department of Chemistry, Texas A&M University, College Station, Texas 77843, United States

## Abstract

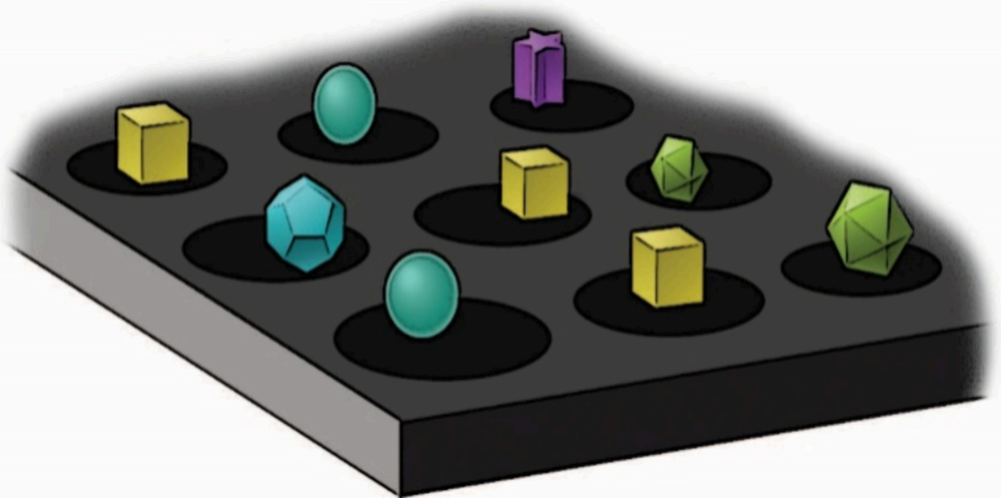

Electrodeposition of nanoparticles is investigated with a multichannel potentiostat in electrochemical and chemical arrays. *De novo* deposition and shape control of palladium nanoparticles are explored in arrays with a two-stage strategy. Initial conditions for electrodeposition of materials are discovered in a first stage and then used in a second stage to logically expand chemical and electrochemical parameters. Shape control is analyzed primarily with scanning electron microscopy. Using this approach, optimized conditions for the electrodeposition of cubic palladium nanoparticles were identified from a set of previously untested electrodeposition conditions. The parameters discovered through the array format were then successfully extrapolated to a traditional bulk three-electrode electrochemical cell. Electrochemical arrays were also used to explore electrodeposition parameters reported in previous bulk studies, further demonstrating the correspondence between the array and bulk systems. These results broadly highlight opportunities for electrochemical arrays, both for discovery and for further investigations of electrodeposition in nanomaterials synthesis.

## 1 Introduction

Electrochemical nanomaterials synthesis (i.e., electrodeposition) expands available synthetic handles to include both electrochemical and chemical parameters.^1^ Specifically, electrodeposition affords additional variation of physicochemical processes, such as electrochemical potentials and mass transport, through control of the potential or current at the electrode/solution interface. Ultimately, this influences precursor reduction and nanoparticle growth, thus providing control of synthetic parameters that are not typically accessed in colloidal nanoparticle synthesis.^1−7^ For example, an oscillating square wave potential can be applied in which growth conditions switch from deposition (more reducing potentials) to dissolution (more oxidizing potentials), affording an opportunity to electrochemically anneal particles at the electrode interface.^3−6^ Reaction potentials can also be fine-tuned over the time course of a nanoparticle growth process to modulate reduction kinetics at key points in the nanoparticle shape development.^2^ In addition, electrochemistry can provide quantitative information about the dynamic chemical environment during nanoparticle growth, thereby guiding synthetic design from fundamental principles.^2,8^ However, a drawback of electrochemical materials synthesis is the inherently serial nature of the approaches typically taken. To screen electrochemical parameters or develop combinatorial approaches with existing platforms is typically difficult to scale, requiring a dedicated potentiostat for each condition. Overcoming the limit on throughput is an important step in accelerating materials discovery with electrochemistry.

Approaches to increase the throughput of electrochemical experiments have been developed,^9−11^ including limited success in materials synthesis and analysis.^12−16^ To address throughput and enable materials discovery via electrochemistry, we developed a *parallel* approach to the electrodeposition of metal nanoparticles, using palladium (Pd) as a proof of concept. This enables development of electrochemical synthesis and discovery with an experimental bandwidth equivalent to colloidal synthesis. We enable discovery with a multichannel potentiostat recently described by Gerroll et al.,^17^ colloquially referred to as “Legion.” Designed around the footprint of a 96-well plate, Legion uses a single, large working electrode (WE) and 96 quasi-reference counter electrodes (QRCEs) to conduct 96 parallel electrochemical experiments, each in one of the 96 separate wells. Instrument control by a field programmable gate array (FPGA) enables independent variation of both the chemical and electrochemical parameters in each well.

**Figure 1:**
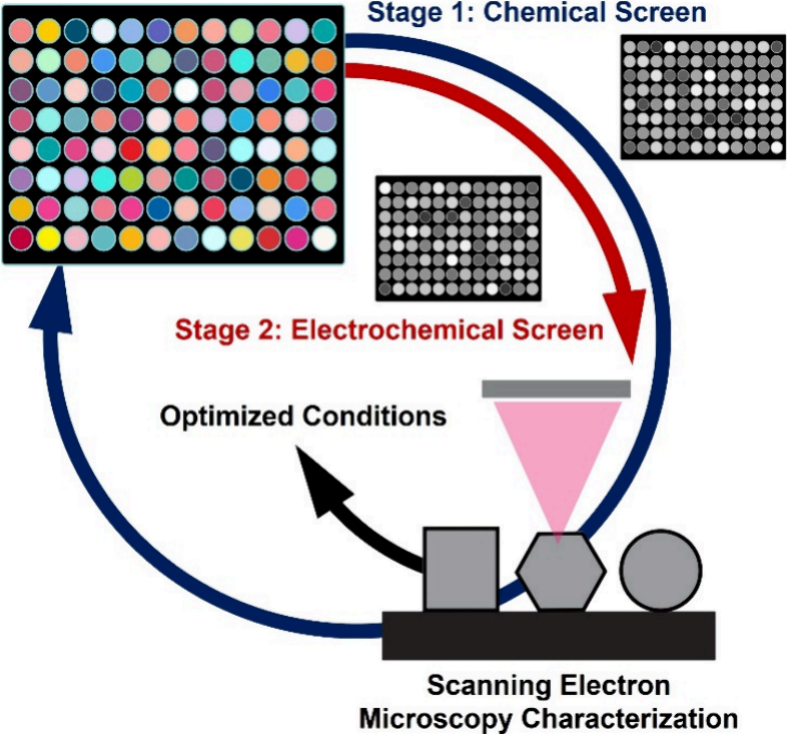
Schematic representation of the experimental workflow for synthesis discovery and optimization via array-based parallel electrodeposition of metal nanoparticles.

Here, we utilize Legion to conduct high-throughput electrochemical materials synthesis in two stages (Figure 1). We first identify chemical components that facilitate shape control in the electrodeposition of polyhedral Pd nanoparticles. Then, in a second stage, we simultaneously optimize the electrochemical parameters and chemical conditions required for the synthesis of a particular shape. For this work, each Legion deposition array was composed of 24 experimental conditions (electrochemical and/or chemical variations of the nanoparticle deposition environment) along with four replicates of each condition, for a total of 96 experiments. For example, conditions varied in a single array included: square wave vs constant potential deposition, deposition potential (or upper and lower potentials for square wave synthesis), surfactant concentration, and metal precursor concentration. In experiments described here, Legion required approximately 4 h to set up and 1 h to run the electrodeposition, followed by time for imaging with scanning electron microscopy (SEM), providing significant acceleration of the electrochemical synthetic discovery process. The time-limiting factor for throughput becomes SEM imaging, as is the case for combinatorial colloidal synthesis development. In a third facet of this work, we demonstrate that optimized synthetic conditions identified with Legion can be translated to a bulk three-electrode setup, completing the bridge to standard electrochemical experiments.

## 2 Results and Discussion

**Figure 2:**
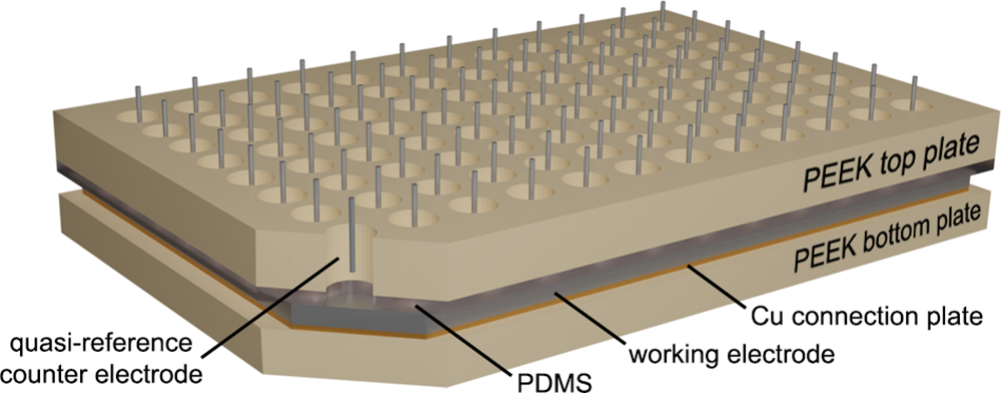
Schematic representation of the Legion 96-well plate design and electrode configuration.

Parallel electrodeposition experiments using Legion were conducted in a 96-well plate with a single glassy carbon WE (110 × 73 mm) and 96 silver (Ag/AgO) QRCEs (Figure 2 and Figure S1). The glassy carbon WE and Ag/AgO QRCEs were polished immediately prior to use. Electrodeposition solutions were prepared separately in 20 mL glass scintillation vials, and 300 μL of the appropriate solution was pipetted into each well of the assembled 96-well plate. Electrochemical parameters of each well are controlled independently in Legion through an FPGA and custom software. Electrodeposition was initiated with an equilibration step of 500 mV for 100 ms, followed by a nucleation step of −200 mV for 100 ms. Continued nanoparticle growth was then carried out for 30 min using either a square wave or constant potential, as described below. Electrodeposition was conducted at room temperature and in the presence of oxygen from the air. Detailed experimental procedures are described in the Experimental Section.

In the first stage of discovery, the chemical composition of the deposition solution was explored at selected electrodeposition parameters. Solutions of tetrachloropalladic (II) acid (H_2_PdCl_4_) precursor along with either perchloric acid (HClO_4_, 0.1M) or an aqueous solution of the quaternary ammonium surfactant hexadecyltrimethylammonium bisulfate (CTAHSO_4_, 0.1M) were prepared. HClO_4_ has been used in the literature as an electrolyte for the electrodeposition of shaped Pd nanoparticles,^18^ and quaternary ammonium surfactants are commonly used in colloidal syntheses. These solutions were arrayed with Legion. The conditions tested in the full 96-well experimental array are shown in Table S1 and the products of these conditions are shown in Figures S2–S4. In this stage, 12 different electrochemical deposition conditions were explored for each solution composition to generate a broad survey of 24 previously unexplored electrodeposition parameters. Electrochemical parameters (referenced vs Ag/AgO) included variation of lower (reducing) and upper (oxidizing) potentials of an applied square wave, frequency of oscillation between potentials of the square wave, and constant potential deposition at a range of potentials. The rapid survey of this wide range of synthetic conditions is not possible using any existing electrodeposition technique.

**Figure 3:**
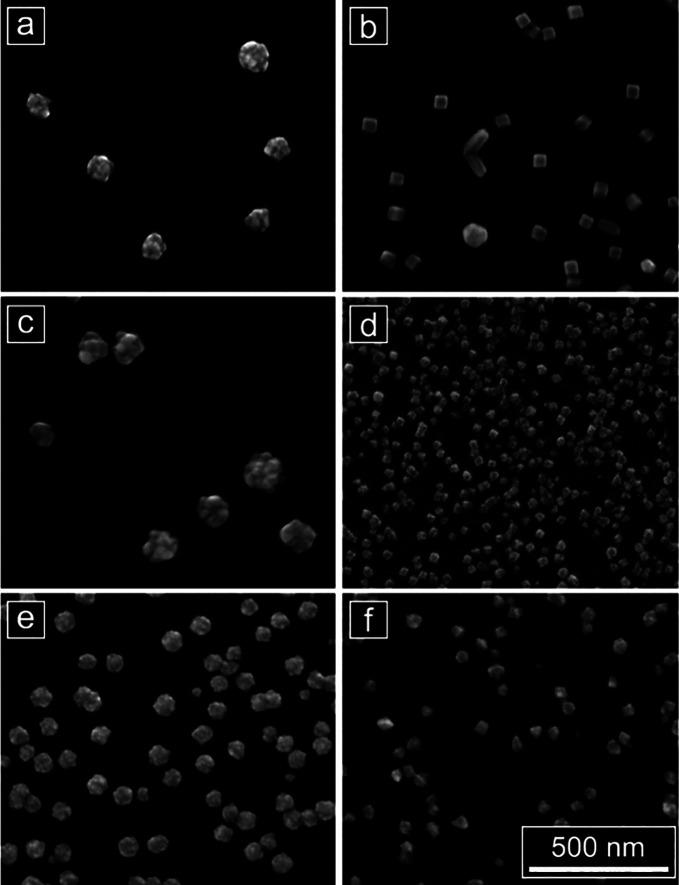
SEM images of Pd nanoparticles synthesized from an H_2_PdCl_4_ precursor in (a, c) 0.1 M HClO_4_ (b, d) 0.1 M CTAHSO_4_, (e) 0.1 M CTAC; or (f) 0.1 M CTAB. Particles in (a) and (b) were electrodeposited using a square wave that oscillated between a lower potential of 0 mV and an upper potential of 500 mV at 100 Hz. Particles in (c), (d), and (f) were electrodeposited at a constant potential of 100 mV, and particles in (e) were electrodeposited at a constant potential of 50 mV. These results identify CTAHSO_4_ as a promising surfactant for enabling shape control in electrodeposition and show the versatility of this parallel electrodeposition approach for simultaneously testing different chemical compositions of the deposition solution and electrodeposition methods (potentials are reported vs Ag/AgO QRCE; scale bar: 500 nm).

From this stage, CTAHSO_4_ was identified as a promising chemical additive for the electrodeposition of nanoparticles with well-defined polyhedral shapes, yielding Pd cubes at multiple deposition conditions tested (Figure 3b,d, Figures S3 and S4f–h). In contrast, deposition in HClO_4_ resulted in particles with poorly defined or “spiky” shapes at all conditions of the array (Figure 3a,c, Figures S2 and S4a–d). Successful cube-forming conditions in CTAHSO_4_ included a square wave deposition with an upper potential (*E*_U_) of 500 mV and a lower potential (*E*_L_) of 0 mV (Figure 3b), as well as constant potential deposition at 100–200 mV (Figure 3d). The “triangular” particles observed to form concurrently with the cubes in CTAHSO_4_ are right bipyramids. These particles have the same {100} surface facets as cubes but have a planar, mirror twin defect and are a commonly observed coproduct in the synthesis of cubic nanoparticles via colloidal approaches.^19^

This initial array revealed parameters of the applied potential that were important in the morphology and quality of the Pd nanoparticles formed in the presence of CTAHSO_4_. For example, in the case of an applied square wave, if *E*_U_ was increased to 600 or 650 mV while *E*_L_ was maintained at 0 mV, no particles formed, thus establishing an upper bound for *E*_U_. Similarly, the product nanoparticles were more rounded and less cubic when the potential in constant potential deposition was increased above 200 mV (Figure S4e–h). The impact of changing the frequency of the square wave from 100 to 25 or 5 Hz was subtle and likely an effect of the capacitance of the macroelectrode used here (Figure S3a–c). Thus, the frequency parameter for square wave deposition was kept constant at 100 Hz in subsequent arrays.

Results from the CTAHSO_4_ array prompted an additional exploration of the chemical composition of the deposition solution. Deposition in two additional surfactants commonly used in colloidal nanoparticle synthesis—hexadecyltrimethylammonium bromide (CTAB, 0.1M) and hexadecyltrimethylammonium chloride (CTAC, 0.1M)—was also probed in a separate array. Electrodeposition in CTAC did not yield well-defined shapes under any deposition condition tested, including constant potential or square wave deposition for a range of potentials (Figure 3e). At the room temperature conditions of this experiment, CTAB had a low solubility and precipitated out of solution over the 30 min electrodeposition period, thus limiting particle growth (Figure 3f).

**Figure 4:**
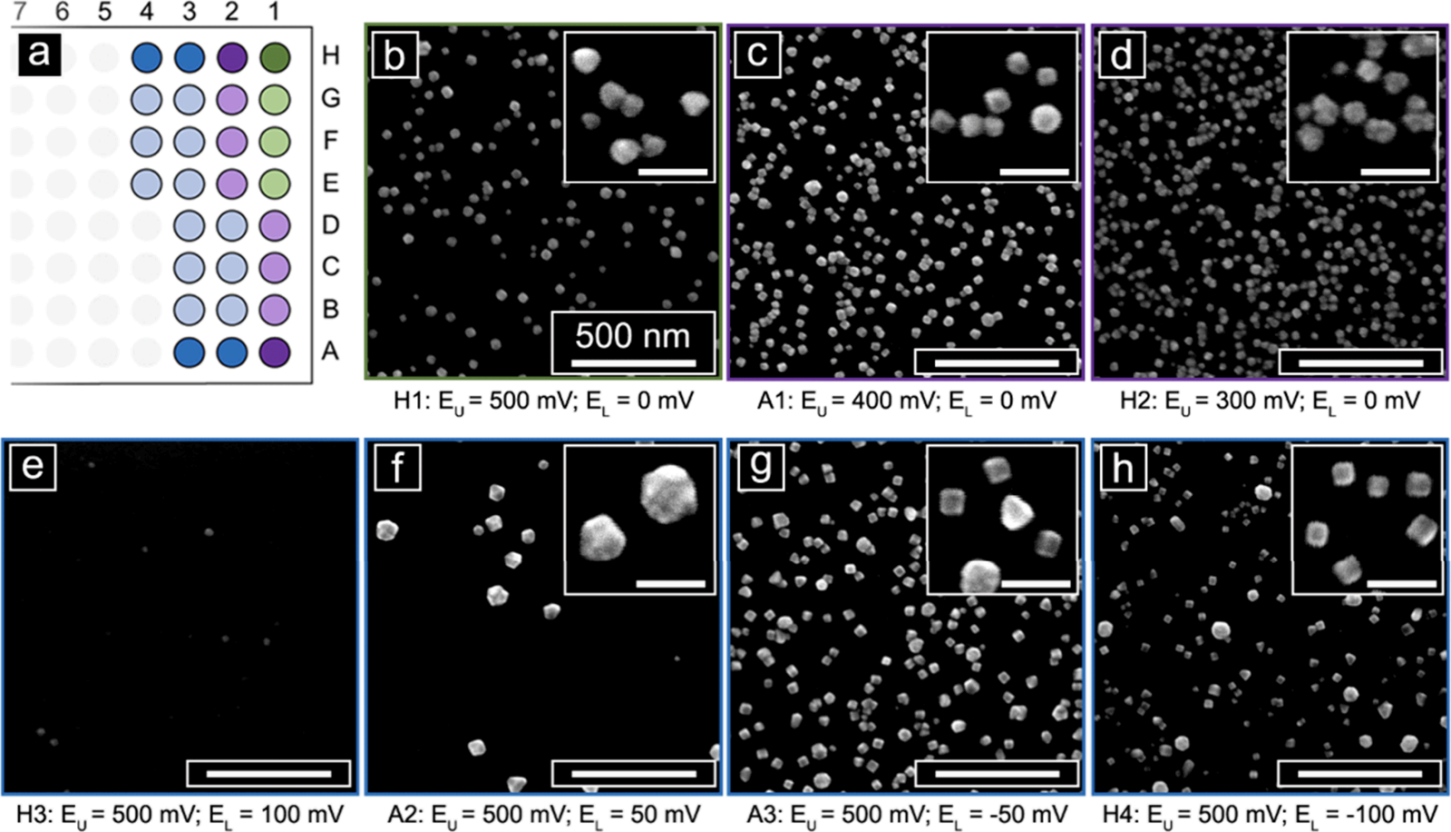
Optimization of square wave Pd cube electrodeposition conditions. (a) Schematic of the 96-well plate format used in the experiment, highlighting the set of wells used for the two experimental gradients shown in (b–h). Seven different conditions were tested, with four replicates of each condition. The wells highlighted in green are part of both gradients. (b–d) SEM images of Pd nanoparticles deposited with an *E*_L_ of 0 mV and *E*_U_ values of (b) 500, (c) 400, and (d) 300 mV. (e–h) SEM images of Pd nanoparticles deposited with an *E*_U_ of 500 mV and *E*_L_ values of (e) 100, (f) 50, (g) −50, and (h) −100 mV (potentials are reported vs Ag/AgO QRCE; major scale bars: 500 nm, inset scale bars: 100 nm).

The first stage of screens of the chemical composition of the deposition solution identified promising conditions. With the goal of improving the quality of the product cubes, we then used Legion in a second stage to tune chemical composition further and to optimize electrodeposition parameters for Pd nanoparticle syntheses in CTAHSO_4_, as shown in Figure 3b,d. Focusing first on the square wave potential, two key parameters to optimize are *E*_U_, at which the growing particles can be oxidized, and *E*_L_, which controls the rate of metal ion reduction and thus nanoparticle growth. These two parameters were expanded in a subset of wells in the array: seven distinct conditions with four replicates each for a total of 28 total wells (Figure 4a). Figure 4b–d shows the effect of changing *E*_U_ to 500, 400, or 300 mV. These results suggest that an oxidative potential of 400 or 500 mV is beneficial for producing cubes (Figure 4b,c), with a potential of 300 mV yielding less well-formed particle shapes (Figure 4d). Similarly, Figure 4b,e–h illustrates the influence of changing *E*_L_. This second set of experiments shows that modifying *E*_L_ to tailor the reduction rate of the Pd precursor has a more significant effect on shape development than changing *E*_U_, which influenced the quality of the shape generated. Optimal *E*_L_ for cube formation was determined to be 0 or −50 mV vs Ag/AgO QRCE (Figures 4b,g). Higher *E*_L_ potentials (50 or 100 mV) yielded fewer particles and particles with truncations (Figure 4e,f), while a lower *E*_L_ potential (−100 mV) yielded larger pseudospheres as a byproduct along with the cubes (Figure 4h).

Optimization for Pd cube deposition in CTAHSO_4_ included additional conditions of deposition potential, variation of the parameters of the nucleation step, decreasing surfactant concentrations, and increasing or decreasing concentrations of H_2_PdCl_4_ (Table S2, Figures S5–S8). Additional conditions were also tested. For example, in the case of constant potential deposition, the optimal potential for cube formation was 100 mV. Decreasing the concentration of surfactant from 100 to 50 mM had minimal effect on particle geometry, but decreasing the surfactant concentration further to 25 or 12.5 mM yielded truncated or rounded shapes (Figure S6). Lowering the concentration of H_2_PdCl_4_ from 0.5 to 0.25 mM resulted in less well-defined particles, while increasing the H_2_PdCl_4_ concentration to 1 mM had a minimal effect on shape (Figure S7). Additional effort to tune particle morphology by modifying the duration or potential of the nucleation step yielded poor quality nanoparticles (Figure S8).

**Figure 5:**
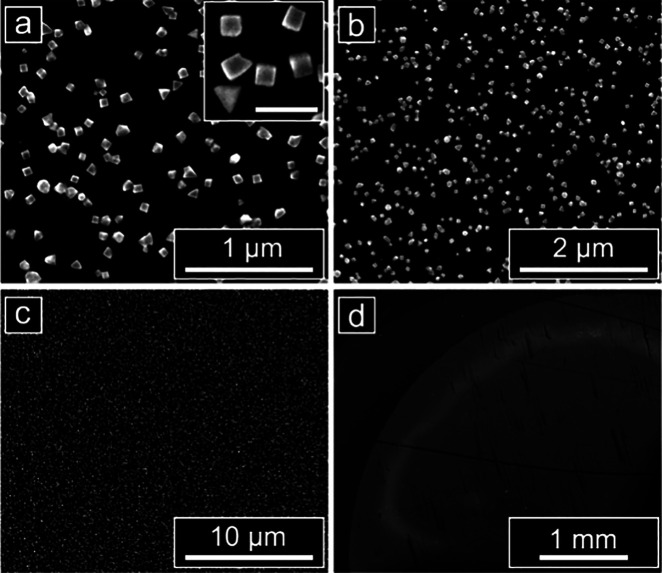
Electrodeposited Pd cubes synthesized with the identified optimal square wave deposition conditions (*E*_U_ = 500 mV, *E*_L_ = −50 mV) and a deposition time of 60 min. The coverage of particles is relatively uniform across the area of the glassy carbon electrode that is exposed in the well (potentials are reported vs Ag/AgO QRCE; major scale bars: (a) 1 μm; (b) 2 μm; (c) 10 μm; (d) 1 mm; inset scale bar: 200 nm).

The second stage of optimization highlighted the importance of the upper and lower potentials of the square wave for tuning Pd nanoparticle shape in this synthesis, with *E*_U_ = 500 mV and *E*_L_ = −50 mV yielding the highest quality cubes. Parallel deposition arrays showed reasonably good consistency across multiple wells with the same deposition conditions and chemical growth solution composition, which as employed here provide built-in replicate experiments as part of the array (Figure S9). These replicates can be used to assess the reproducibility of a particular deposition condition and to identify any outliers. Along with these particle growth conditions, a nucleation step of −200 mV for 100 ms was optimal for achieving well-formed particles in the subsequent square wave step. Additional investigation of the chemical composition of the growth solution in this stage revealed that 0.5–1.0 mM H_2_PdCl_4_ and 50–100 mM CTAHSO_4_ produced the most well-defined cubes. Together these insights suggest that controlling the reduction rate of the Pd precursor through a combination of H_2_PdCl_4_ concentration and *E*_L_ drives the formation of the cubes, which is consistent with principles of kinetically controlled growth from colloidal nanoparticle synthesis.^20,21^ In addition, even though stabilization of the nanoparticles against aggregation is not required due to their immobilization on the electrode surface, the need for a minimum concentration of CTAHSO_4_ to achieve well-formed cubes suggests a role of this surfactant in modulating the reduction rate at the nanoparticle surface. The optimal conditions identified from the array described above were modified further by increasing the deposition time to 60 min in a subsequent array, yielding larger, well-defined cubes (Figure 5). The particles deposit with a high and relatively uniform coverage across the area exposed in each well. Changing the H_2_PdCl_4_ precursor concentration from 0.25 to 1.0 mM in 0.25 mM increments while using a 60 min electrodeposition duration increased the density of Pd cubes on the electrode surface, providing control of coverage independent from shape (Figure S10).

**Figure 6:**
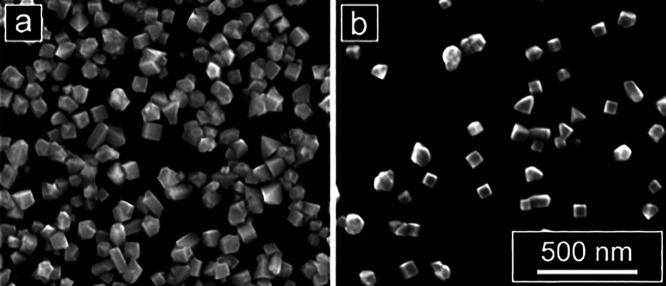
Translation of the optimized Pd cube synthesis conditions identified with Legion to a bulk three-electrode cell: (a) Pd nanoparticles electrodeposited in a bulk cell (Ag/AgCl || Pt) and (b) Pd nanoparticles electrodeposited using analogous conditions in Legion (Ag/AgO QRCE). All deposition potentials were shifted +193 mV to account for the difference in reference electrode potentials between Ag/AgCl and Ag/AgO (scale bar: 500 nm.)

Translating synthetic conditions and parameters identified using the unique cell geometry of Legion to a more standard bulk electrochemical cell with a three-electrode geometry is important to establish the generalizability and broader relevance of this parallel synthetic development approach undertaken here for discovery. Thus, we directly translated the optimized synthetic conditions for Pd cubes identified with Legion to electrodeposition in a bulk cell with 10 mL of reaction solution, a glassy carbon electrode, a Ag/AgCl reference electrode (RE), and a platinum (Pt) wire counter electrode. The open circuit potential difference between a Ag/AgO QRCE and a Ag/AgCl RE was measured to be 193 mV, and therefore, all potentials were increased by 193 mV for the bulk electrodeposition relative to the potentials used for electrodeposition with Legion. These conditions yielded the formation of cubes in the bulk cell, along with the corresponding twinned right bipyramids as observed in Legion (among other shapes, discussed below), confirming that Legion can be used as a rapid screening tool to significantly streamline identification of conditions for electrochemical materials synthesis that can then be implemented in standard bulk cells (Figure 6).

Parameters identified using Legion then need to be fine-tuned in the bulk cell to account for differences between the Legion and bulk systems, such as cell geometry, electrode surface area, and electrode capacitance. For example, in the bulk cell, more rods and poorly formed side products are generated alongside the cubes and bipyramids than are observed in Legion, and the overall density of particles on the electrode surface is also higher (Figure 6a). This suggests that there are slight differences in the nucleation of particles on the two different GC electrodes. The number and density of nucleation sites can influence the diffusion of metal ions at the surface, possibly leading to an increased generation of side products in the bulk cell as a consequence of an increased coverage of particles. The effects of particle density on electrodeposition of nanomaterials have been described in detail by Penner.^22^ The surface area of the bulk GC electrode (19.6 mm^2^) is about half of the area of the GC electrode exposed in a single Legion well (38.5 mm^2^). In addition, while the concentration of H_2_PdCl_4_ is constant between the two systems, the total amount of available Pd^2+^ ions is higher in the bulk cell due to the increased volume (10 mL vs 0.3 mL). We also found that stirring of the growth solution at 200 rpm was required to achieve significant electrodeposition in the bulk cell. Without stirring, a low density of very small particles formed. The differences between the two systems can be accounted for by optimizing parameters such as *E*_nuc_ and [H_2_PdCl_4_] in the bulk cell to control the rate and extent of nucleation. Further, while the area of the GC electrode exposed in each Legion well is only slightly larger than the area of the bulk electrode, the total size of the Legion GC electrode plate is much larger, and thus, the Legion electrodes will have a higher capacitance. Consideration of capacitance is particularly important for square wave deposition where the potential is oscillated between *E*_L_ and *E*_U_ at a high frequency (100 Hz) as well as for short pulses such as *E*_nuc_ (100 ms). Slightly decreasing the duration of the nucleation step or increasing *E*_nuc_ in the bulk cell could help account for the effect of capacitance on the nucleation step, while decreased frequencies of oscillation between *E*_U_ and *E*_L_ in Legion will better correspond to higher frequencies in the bulk cell. A decreased nucleation step duration in the bulk cell would more accurately match the amount of time Legion spends at the set *E*_nuc_ potential as a result of any capacitance limitations, while an increased *E*_nuc_ in the bulk cell would compensate for situations where Legion does not have time to reach the value of *E*_nuc_ that was programmed.

**Figure 7:**
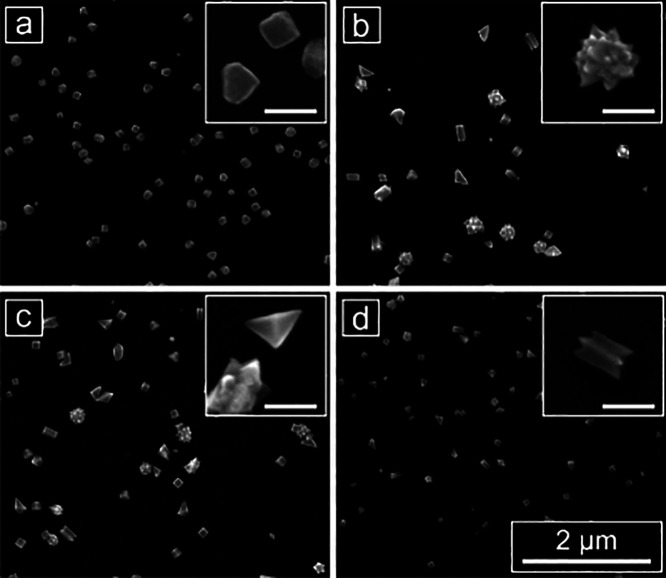
SEM images of the shape-tuning effect of doping different amounts of bromide ions (as NaBr) into the electrodeposition solution in a Legion experiment: (a) No NaBr; (b) 5 μM NaBr; (c) 10 μM NaBr; (d) 100 μM NaBr. Insets in (b–d) show representative examples of particle shapes observed across all three conditions (major scale bar: 2 μm; inset scale bars: 200 nm).

Results from bulk cell electrodeposition can also be translated directly to Legion, where expanded solution conditions can be easily varied. In a previous study, McDarby et al. studied the effect of bromide ions on electrochemical Pd nanoparticle deposition and correlated results from electrochemical deposition with those observed in colloidal nanoparticle growth.^2^ Thus, we examined the influence of doping bromide ions (as NaBr) into the chemical growth solution used for the synthesis of the cubes in a Legion array as a comparative benchmark. Starting from the deposition solution optimized above for cube formation, the concentration of bromide was increased from 0 μM—the condition for cube synthesis—to 5, 10, or 100 μM across wells of a Legion array while keeping all other parameters of the cube deposition constant. Doped bromide was present in the reaction solution at the start of the electrodeposition process. Adding bromide changed the product nanostructures from cubes to a mixture of pentatwinned rods with fins, stellated icosahedra, and large bipyramid structures (Figure 7). These shapes are analogous to products observed previously with the addition of bromide,^2,23^ except that they do not have corrugated surfaces—likely because the concentration of chloride ions is lower in the Legion experiment (2 mM vs 14.5 mM). This experiment provides a clear link between the array-based electrodeposition platform using Legion with an Ag/AgO QRCE and a more standard three-electrode cell with a single glassy carbon working electrode, a Pt wire counter electrode, and a Ag/AgCl reference electrode. The nanoparticle growth potentials differ between the two experiments to compensate for the different reference electrodes. The ability to form analogous nanoparticles in both standard- and array-based electrodeposition approaches—as well as in colloidal nanoparticle growth—highlights the promise of the Legion platform for translatable materials synthesis discovery.

## 3 Conclusions

Parallel electrodeposition of nanomaterials with Legion successfully combines the flexibility and enhanced parameter space of electrochemical materials synthesis with the rapid combinatorial screening capabilities normally characteristic of colloidal nanoparticle synthesis. As demonstrated here, the array approach undertaken provides a route to discover initial conditions for electrodeposition of materials in a first stage and then is used in a second stage to logically expand chemical and electrochemical parameters. We further directly link results from Legion to more standard electrodeposition setups and vice versa. Looking ahead, this approach provides a platform for future experiments that couple arrays of electrodeposited nanoparticles with electrocatalysis experiments in Legion to test performance as a function of arrayed nanomaterial structure or arrayed electrocatalysis conditions.

A limiting factor on throughput for this platform is now the materials characterization step—here, SEM—as is the case for colloidal nanoparticle synthesis methods. This points to a need for methods of automating SEM imaging as well as the development of tools for nanomaterials characterization that do not require electron microscopy but still capture important parameters such as shape, size, and dispersity. The regular and predictable arrangement of nanoparticle samples deposited on the electrode surface due to the uniform spacing of the 96 wells, combined with the highly planar nature of the glassy carbon electrode, provide a possible opportunity for interfacing automated SEM imaging tools such as those used in critical dimension SEM measurements in the semiconductor industry. There is a correlated need for advances in automated image analysis to extract quantitative information regarding nanoparticle shape, size, and dispersity from such large data sets of images. Machine learning will likely play an important role in facilitating this analysis, although significant development is still required to enable the automated classification of three-dimensional shapes in SEM images.

## 4 Experimental Section

### 4.1 Materials

Palladium(II) chloride (PdCl_2_, 99.9%, metals basis), hydrochloric acid solution (HCl, 1N), sodium bromide (NaBr, 99.99%, metals basis), perchloric acid (HClO_4_), concentrated hydrochloric acid (HCl, ACS grade), and concentrated nitric acid (HNO_3_, ACS grade) were purchased from Thermo Scientific Chemicals. Hexadecyltrimethylammonium hydrogen sulfate (CTAHSO_4_, >98%) and hexadecyltrimethylammonium chloride (CTAC, ≥95.0%) were purchased from Tokyo Chemical Industries (TCI). Hexadecyltrimethylammonium bromide (CTAB, BioXtra, ≥99%) was purchased from MilliporeSigma. Concentrated sulfuric acid (H_2_SO_4_, 95–98%, ACS grade) was purchased from VWR. All reagents were used as received. Tetrachloropalladic acid (H_2_PdCl_4,_ 100 mM solution) was prepared by combining 0.354 g of PdCl_2_ and 20 mL of 0.2 M HCl in a 50 mL round-bottom flask. The solution was capped and stirred for 3 h until the solid fully dissolved and the solution became a clear, dark orange color. Concentrated H_2_SO_4_ was diluted to 100 mM by adding acid dropwise to deionized (DI) water over ice (caution: strong acid). All solutions were prepared with DI water (18.2 MΩ resistivity, Millipore Direct–Q3, and MilliporeSigma Milli-Q IQ 7000).

### 4.2 Legion Electrodes

A glassy carbon plate (redox.me, 110 × 73 × 3 mm) was used as a working electrode, and 25 mm long pieces of silver (Ag) wire (Millipore Sigma, 1 mm diameter, 99.9%) served as the quasi-reference counter electrodes (QRCEs). Prior to use, the glassy carbon electrode was successively polished on a MasterTex Buehler polishing pad with alumina powder slurries of decreasing size (0.3 and 0.05 μm MicroPolish Alumina, Buehler), followed by polishing on the bare polishing pad with DI water only. The electrode was rinsed with DI water and sonicated in DI water between each polishing step. The Ag wire QRCEs were lightly sanded and rinsed with water and ethanol prior to use. It is important that the electrodes are polished the same day as the electrodeposition.

### 4.3 Legion Instrument Design

The layout of the wells in the Legion multichannel potentiostat is based on a 96-well microtiter plate. The Legion well plate assembly is composed of a polyether ether ketone (PEEK) top plate with a 12 × 8 array of 7 mm diameter circular openings, a polydimethylsiloxane (PDMS) gasket with the same array of circular openings, the glassy carbon plate working electrode, a copper base plate to ensure conductivity across the working electrode, a second PDMS gasket, and a continuous bottom PEEK plate. The assembly is then joined together by eight machine screws—one in each corner and one at the center of each edge of the plate—with the compressed PDMS gaskets forming a seal around each well. The exposed glassy carbon electrode area in each well is 38.5 mm^2^, defined by the area of each opening in the PEEK top plate. The electroactive surface area of each QRCE is approximately 35 mm^2^. QRCEs in each column of the array are connected to an eight-channel potentiostat board. The 12 eight-channel potentiostat boards interface with a field programmable gate array (FPGA) and custom software, enabling independent control of the QRCE in each well. Additional details about the Legion instrument design can be found in ref (17).

### 4.4 Legion Electrodeposition Solutions

Solutions for electrodeposition were prepared in 20 mL glass scintillation vials, and 300 μL of the prepared solution was then added to the appropriate well in the Legion well plate. To prepare a 0.5 mM solution of H_2_PdCl_4_ in 0.1 M CTAHSO_4_, as used in the optimized electrodeposition of Pd cubes, 100 μL of an aqueous stock solution of 0.1 M (100 mM) H_2_PdCl_4_ was added to 20 mL of an aqueous stock solution of 0.1 M CTAHSO_4_. Other solutions were prepared in a similar manner, adjusting stock solution concentrations and volumes as necessary to achieve the overall concentrations of each reagent listed in the main text. While the combined electrodeposition solutions were prepared fresh on the same day the electrodeposition was conducted, the separate stock solutions of 0.1 M H_2_PdCl_4_ and 0.1 M CTAHSO_4_ can be stored for at least 6 months.

### 4.5 Parallel Electrodeposition of Pd Nanoparticles Using Legion

Unless otherwise specified, each electrodeposition of Pd nanoparticles began with a brief equilibration at 500 mV for 100 ms, followed by a nucleation step of −200 mV for 100 ms to generate small nanoparticle “seeds” on the electrode surface (all potentials vs Ag/AgO QRCE). Continued nanoparticle growth was then carried out for 30 or 60 min using either a square wave potential (oscillating between a more oxidizing potential and a more reducing potential) or a constant potential. All electrodeposition experiments were conducted at room temperature.

Once electrodeposition was complete, the Legion well plate was disassembled and the glassy carbon working electrode was rinsed with DI water to remove residual surfactant and other reagents. The working electrode was then allowed to dry in air at room temperature overnight prior to being imaged by scanning electron microscopy (SEM). All other components of the well plate were washed with soap and water, rinsed with ethanol, and left to dry. The Ag/AgO QRCEs were rinsed with water and ethanol and left to dry.

### 4.6 Bulk Cell Electrodeposition of Shaped Palladium Nanoparticles

Bulk cell investigations were conducted in a five-port glass electrochemical cell (Gamry Instruments Dr. Bob’s Cell) with a glassy carbon electrode (GCE) as the working electrode (5 mm OD × 4 mm thick disk insert, Pine Research), a platinum (Pt) wire as the counter electrode (Gamry Instruments, 0.406 mm diameter), a Ag/AgCl reference electrode (RE) (4 M KCl, Koslow Scientific), and a stir bar (10 mm × 3 mm). GCEs were prepared by polishing with a 0.05 μm alumina polishing powder slurry (MicroPolish Alumina, Buehler) on a MasterTex Buehler polishing pad, sonicating in DI water, and rinsing before being inserted into a poly(tetrafluoroethylene) (PTFE) electrode tip holder (Pine Research). Once assembled, the electrode in the PTFE tip holder was rinsed and dried with air. The RE was isolated from the reaction solution by a bridge tube with a glass frit containing 100 mM sulfuric acid to stop leakage of chloride and to prevent damage to the reference electrode.

The assembled cell was clamped above a stir plate, and electrode leads were connected to the potentiostat (Gamry Instruments 1010B or 1010E). To a prepared cell, 10 mL of a growth solution containing 0.5 mM H_2_PdCl_4_ in 100 mM CTAHSO_4_ (described above) was added and stirred at 200 rpm for 10 min before beginning electrodeposition. The electrodeposition of Pd nanoparticles began with a brief equilibration at 693 mV for 100 ms, followed by a nucleation step of −7 mV for 100 ms to generate small nanoparticle “seeds” on the electrode surface (all potentials vs Ag/AgCl). A square wave potential oscillating at a 100 Hz frequency between a more oxidizing potential, *E*_U_ = 693 mV, and a more reducing potential, *E*_L_ = 143 mV, was then applied for 60 min. All electrodeposition experiments were conducted at room temperature, and stirring at 200 rpm was continued throughout.

Stir bars, bridge tube frits, glassware, and PTFE electrode tip holders were cleaned with aqua regia (1:3 ratio of concentrated nitric acid: concentrated hydrochloric acid; caution: strong acid) and DI water between uses. After use, reference electrodes were rinsed with 40 °C DI water and returned to an electrode storage solution (premade potassium hydrogen phthalate and potassium chloride solution in water, Fisher Chemical/ThermoFisher).

### 4.7 Electron Microscopy

The deposited nanoparticles synthesized using Legion were characterized using an FEI Quanta 600 field emission SEM. A custom sample holder consisting of a machined aluminum rectangular well and plastic set screws was constructed to hold the glassy carbon working electrode in the SEM (Figure S1D). Plastic set screws are important so that the electrode is not scratched or cracked. Conductive copper tape was used as a marker on the sample holder to identify the orientation of the glassy carbon electrode in the SEM.

The deposited nanoparticles synthesized in the bulk electrochemical cell were characterized using an FEI Quanta 650 field emission SEM. Electrodeposited particles were washed 3–4 times with 40 °C DI water and placed in a custom sample holder made of machined aluminum and metal set screws. Samples were dried for at least 30 min under ambient conditions before characterization by SEM.

## Supporting Information Available

Conditions of electrodeposition arrays and additional SEM images (PDF)

## References

[ref1] McDarby S. P.; Personick M. L. Potential-Controlled (R)Evolution: Electrochemical Synthesis of Nanoparticles with Well-Defined Shapes. ChemNanoMat 2022, 8, e202100472 10.1002/cnma.202100472.

[ref2] McDarby S. P.; Wang C. J.; King M. E.; Personick M. L. An Integrated Electrochemistry Approach to the Design and Synthesis of Polyhedral Noble Metal Nanoparticles. J. Am. Chem. Soc. 2020, 142, 21322–21335. 10.1021/jacs.0c07987. 33237754

[ref3] Tian N.; Zhou Z. Y.; Sun S. G.; Ding Y.; Wang Z. L. Synthesis of Tetrahexahedral Platinum Nanocrystals with High-Index Facets and High Electro-Oxidation Activity. Science 2007, 316, 732–735. 10.1126/science.1140484. 17478717

[ref4] Li Y.-Y.; Liao H.-G.; Rao L.; Jiang Y.-X.; Huang R.; Zhang B.-W.; He C.-L.; Tian N.; Sun S.-G. Shape Evolution of Platinum Nanocrystals by Electrochemistry. Electrochim. Acta 2014, 140, 345–351. 10.1016/j.electacta.2014.04.147.

[ref5] Jiang Y.-C.; Mao Y.-J.; Zou J.; Wang H.-H.; Liu F.; Wei Y.-S.; Sheng T.; Zhao X.-S.; Wei L. Electrochemically Shape-Controlled Synthesis of Great Stellated Dodecahedral Au Nanocrystals with High-Index Facets for Nitrogen Reduction to Ammonia. Chem. Commun. 2020, 56, 12162–12165. 10.1039/D0CC04326E. 32909571

[ref6] Mao Y.-J.; Liu F.; Chen Y.-H.; Jiang X.; Zhao X.-S.; Sheng T.; Ye J.-Y.; Liao H.-G.; Wei L.; Sun S.-G. Enhancing Electrocatalytic Nitrogen Reduction to Ammonia with Rare Earths (La, Y, and Sc) on High-Index Faceted Platinum Alloy Concave Nanocubes. J. Mater. Chem. A 2021, 9, 26277–26285. 10.1039/D1TA05515A.

[ref7] Siegfried M. J.; Choi K.-S. Electrochemical Crystallization of Cuprous Oxide with Systematic Shape Evolution. Adv. Mater. 2004, 16, 1743–1746. 10.1002/adma.200400177.

[ref8] Halford G. C.; Personick M. L. Bridging Colloidal and Electrochemical Nanoparticle Growth with *In Situ* Electrochemical Measurements. Acc. Chem. Res. 2023, 56, 1228–1238. 10.1021/acs.accounts.3c00112. 37140656

[ref9] Chen H. J.; Mo Y. M. Accelerated Electrosynthesis Development Enabled by High-Throughput Experimentation. Synthesis-Stuttgart 2023, 55, 2817–2832. 10.1055/a-2072-2617.

[ref10] Wiebe A.; Gieshoff T.; Möhle S.; Rodrigo E.; Zirbes M.; Waldvogel S. R. Electrifying Organic Synthesis. Angew. Chem., Int. Ed. 2018, 57, 5594–5619. 10.1002/anie.201711060. PMC5969240 29292849

[ref11] Wills A. G.; Charvet S.; Battilocchio C.; Scarborough C. C.; Wheelhouse K. M. P.; Poole D. L.; Carson N.; Vantourout J. C. High-Throughput Electrochemistry: State of the Art, Challenges, and Perspective. Org. Process Res. Dev. 2021, 25, 2587–2600. 10.1021/acs.oprd.1c00167.

[ref12] Alden S. E.; Siepser N. P.; Patterson J. A.; Jagdale G. S.; Choi M.; Baker L. A. Array Microcell Method (AMCM) for Serial Electroanalysis. Chemelectrochem 2020, 7, 1084–1091. 10.1002/celc.201901976. 36588586 PMC9798888

[ref13] Jiang R. Combinatorial Electrochemical Cell Array for High Throughput Screening of Micro-Fuel-Cells and Metal/Air Batteries. Rev. Sci. Instrum. 2007, 78, 072209 10.1063/1.2755439. 17672740

[ref14] Ley C.; Zengin Çekiç S.; Kochius S.; Mangold K. M.; Schwaneberg U.; Schrader J.; Holtmann D. An Electrochemical Microtiter Plate for Parallel Spectroelectrochemical Measurements. Electrochim. Acta 2013, 89, 98–105. 10.1016/j.electacta.2012.10.151.

[ref15] Lin X. M.; Zheng L. Y.; Gao G. M.; Chi Y. W.; Chen G. N. Electrochemiluminescence Imaging-Based High-Throughput Screening Platform for Electrocatalysts Used in Fuel Cells. Anal. Chem. 2012, 84, 7700–7707. 10.1021/ac300875x. 22946551

[ref16] Roberts M. R.; Spong A. D.; Vitins G.; Owen J. R. High Throughput Screening of the Effect of Carbon Coating in LiFePO_4_ Electrodes. J. Electrochem. Soc. 2007, 154, A921–A928. 10.1149/1.2763968.

[ref17] Gerroll B. H. R.; Kulesa K. M.; Ault C. A.; Baker L. A. Legion: An Instrument for High-Throughput Electrochemistry. ACS Meas Sci. Au 2023, 3, 371–379. 10.1021/acsmeasuresciau.3c00022. 37868360 PMC10588931

[ref18] Tian N.; Zhou Z. Y.; Yu N. F.; Wang L. Y.; Sun S. G. Direct Electrodeposition of Tetrahexahedral Pd Nanocrystals with High-Index Facets and High Catalytic Activity for Ethanol Electrooxidation. J. Am. Chem. Soc. 2010, 132, 7580–7581. 10.1021/ja102177r. 20469858

[ref19] Wiley B. J.; Xiong Y.; Li Z.-Y.; Yin Y.; Xia Y. Right Bipyramids of Silver: A New Shape Derived from Single Twinned Seeds. Nano Lett. 2006, 6, 765–768. 10.1021/nl060069q. 16608280

[ref20] Personick M. L.; Mirkin C. A. Making Sense of the Mayhem Behind Shape Control in the Synthesis of Gold Nanoparticles. J. Am. Chem. Soc. 2013, 135, 18238–18247. 10.1021/ja408645b. 24283259

[ref21] Robertson D. D.; Personick M. L. Growing Nanoscale Model Surfaces to Enable Correlation of Catalytic Behavior across Dissimilar Reaction Environments. Chem. Mater. 2019, 31, 1121–1141. 10.1021/acs.chemmater.8b04595.

[ref22] Penner R. M. Mesoscopic Metal Particles and Wires by Electrodeposition. J. Phys. Chem. B 2002, 106, 3339–3353. 10.1021/jp013219o.

[ref23] King M. E.; Personick M. L. Defects by Design: Synthesis of Palladium Nanoparticles with Extended Twin Defects and Corrugated Surfaces. Nanoscale 2017, 9, 17914–17921. 10.1039/C7NR06969C. 29124271

