# Supplementary material for: Nanomaterials Synthesis Discovery via Parallel Electrochemical Deposition
Source: Chem Mater. 2024 Mar 14;36(6):3034–41. doi: 10.1021/acs.chemmater.4c00318 (PMC10976633; doi:10.1021/acs.chemmater.4c00318)
Supplement: Supplementary file 1 — cm4c00318_si_001.pdf [file cm4c00318_si_001.pdf]

Supporting Information for

Nanomaterials Synthesis Discovery via Parallel Electrochemical Deposition

Michelle L. Personick[1,2*]; Abdoulie A. Jallow[3]; Gabriel C. Halford[1]; and Lane A. Baker[3]*

[1] Department of Chemistry, University of Virginia, Charlottesville, Virginia 22904, United States
[2] Department of Chemistry, Wesleyan University, Middletown, Connecticut 06459, United States
[3] Department of Chemistry, Texas A&M University, College Station, Texas 77843, United States

*Corresponding author emails: mpersonick@virginia.edu; lane.baker@chem.tamu.edu

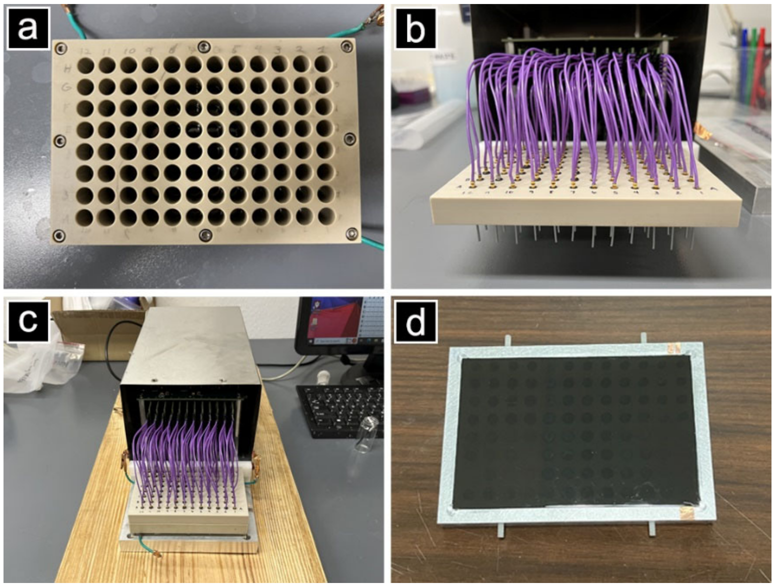

Figure S1. Photographs of the Legion experimental setup (a-c): (a) assembled 96-well plate with glassy carbon electrode at the bottom of the wells; (b) quasi-reference counter electrodes and leads hooked to the FPGA; and (c) fully assembled Legion instrument with field programmable gate array to control the potential in each well. (d) Photograph of the glassy carbon electrode in a custom holder for SEM imaging. Dark circles are areas that comprise the bottom of each well where electrodeposition occurred.

Table 1. Conditions of Screening Array for Pd Electrodeposition in $CTAHSO_4$ Surfactant vs. $HClO_4$

	12	11	10	9	8	7	6	5	4	3	2	1
	For all, unless otherwise specified:<br>E_{equil} = 500 mV for 100 ms; E_{nuc} = -200 mV for 100 ms<br>Frequency = 100 Hz; Deposition Time = 30 min<br>All potentials vs. Ag/AgO QRCE											
	0.5 mM H_2PdCl_4 in 100 mM $CTAHSO_4$						0.2 mM H_2PdCl_4 in 100 mM $HClO_4$					
**H**	$E_L = E_U$<br>= 150 mV	$E_L = E_U$<br>= 250 mV	E_L = 0 mV<br>E_U = 600 mV<br>100 Hz	E_L = 0 mV<br>E_U = 550 mV<br>5 Hz	E_L = 0 mV<br>E_U = 550 mV<br>25 Hz	E_L = 0 mV<br>E_U = 550 mV<br>100 Hz	$E_L = E_U$<br>= 150 mV	$E_L = E_U$<br>= 250 mV	E_L = 0 mV<br>E_U = 600 mV<br>100 Hz	E_L = 0 mV<br>E_U = 550 mV<br>5 Hz	E_L = 0 mV<br>E_U = 550 mV<br>25 Hz	E_L = 0 mV<br>E_U = 550 mV<br>100 Hz
**G**	$E_L = E_U$<br>= 150 mV	$E_L = E_U$<br>= 250 mV	E_L = 0 mV<br>E_U = 600 mV<br>100 Hz	E_L = 0 mV<br>E_U = 550 mV<br>5 Hz	E_L = 0 mV<br>E_U = 550 mV<br>25 Hz	E_L = 0 mV<br>E_U = 550 mV<br>100 Hz	$E_L = E_U$<br>= 150 mV	$E_L = E_U$<br>= 250 mV	E_L = 0 mV<br>E_U = 600 mV<br>100 Hz	E_L = 0 mV<br>E_U = 550 mV<br>5 Hz	E_L = 0 mV<br>E_U = 550 mV<br>25 Hz	E_L = 0 mV<br>E_U = 550 mV<br>100 Hz
**F**	$E_L = E_U$<br>= 150 mV	$E_L = E_U$<br>= 250 mV	E_L = 0 mV<br>E_U = 600 mV<br>100 Hz	E_L = 0 mV<br>E_U = 550 mV<br>5 Hz	E_L = 0 mV<br>E_U = 550 mV<br>25 Hz	E_L = 0 mV<br>E_U = 550 mV<br>100 Hz	$E_L = E_U$<br>= 150 mV	$E_L = E_U$<br>= 250 mV	E_L = 0 mV<br>E_U = 600 mV<br>100 Hz	E_L = 0 mV<br>E_U = 550 mV<br>5 Hz	E_L = 0 mV<br>E_U = 550 mV<br>25 Hz	E_L = 0 mV<br>E_U = 550 mV<br>100 Hz
**E**	$E_L = E_U$<br>= 150 mV	$E_L = E_U$<br>= 250 mV	E_L = 0 mV<br>E_U = 600 mV<br>100 Hz	E_L = 0 mV<br>E_U = 550 mV<br>5 Hz	E_L = 0 mV<br>E_U = 550 mV<br>25 Hz	E_L = 0 mV<br>E_U = 550 mV<br>100 Hz	$E_L = E_U$<br>= 150 mV	$E_L = E_U$<br>= 250 mV	E_L = 0 mV<br>E_U = 600 mV<br>100 Hz	E_L = 0 mV<br>E_U = 550 mV<br>5 Hz	E_L = 0 mV<br>E_U = 550 mV<br>25 Hz	E_L = 0 mV<br>E_U = 550 mV<br>100 Hz
**D**	$E_L = E_U$<br>= 100 mV	$E_L = E_U$<br>= 200 mV	E_L = 0 mV<br>E_U = 650 mV<br>100 Hz	E_L = 0 mV<br>E_U = 500 mV<br>5 Hz	E_L = 0 mV<br>E_U = 500 mV<br>25 Hz	E_L = 0 mV<br>E_U = 500 mV<br>100 Hz	$E_L = E_U$<br>= 100 mV	$E_L = E_U$<br>= 200 mV	E_L = 0 mV<br>E_U = 650 mV<br>100 Hz	E_L = 0 mV<br>E_U = 500 mV<br>5 Hz	E_L = 0 mV<br>E_U = 500 mV<br>25 Hz	E_L = 0 mV<br>E_U = 500 mV<br>100 Hz
**C**	$E_L = E_U$<br>= 100 mV	$E_L = E_U$<br>= 200 mV	E_L = 0 mV<br>E_U = 650 mV<br>100 Hz	E_L = 0 mV<br>E_U = 500 mV<br>5 Hz	E_L = 0 mV<br>E_U = 500 mV<br>25 Hz	E_L = 0 mV<br>E_U = 500 mV<br>100 Hz	$E_L = E_U$<br>= 100 mV	$E_L = E_U$<br>= 200 mV	E_L = 0 mV<br>E_U = 650 mV<br>100 Hz	E_L = 0 mV<br>E_U = 500 mV<br>5 Hz	E_L = 0 mV<br>E_U = 500 mV<br>25 Hz	E_L = 0 mV<br>E_U = 500 mV<br>100 Hz
**B**	$E_L = E_U$<br>= 100 mV	$E_L = E_U$<br>= 200 mV	E_L = 0 mV<br>E_U = 650 mV<br>100 Hz	E_L = 0 mV<br>E_U = 500 mV<br>5 Hz	E_L = 0 mV<br>E_U = 500 mV<br>25 Hz	E_L = 0 mV<br>E_U = 500 mV<br>100 Hz	$E_L = E_U$<br>= 100 mV	$E_L = E_U$<br>= 200 mV	E_L = 0 mV<br>E_U = 650 mV<br>100 Hz	E_L = 0 mV<br>E_U = 500 mV<br>5 Hz	E_L = 0 mV<br>E_U = 500 mV<br>25 Hz	E_L = 0 mV<br>E_U = 500 mV<br>100 Hz
**A**	$E_L = E_U$<br>= 100 mV	$E_L = E_U$<br>= 200 mV	E_L = 0 mV<br>E_U = 650 mV<br>100 Hz	E_L = 0 mV<br>E_U = 500 mV<br>5 Hz	E_L = 0 mV<br>E_U = 500 mV<br>25 Hz	E_L = 0 mV<br>E_U = 500 mV<br>100 Hz	$E_L = E_U$<br>= 100 mV	$E_L = E_U$<br>= 200 mV	E_L = 0 mV<br>E_U = 650 mV<br>100 Hz	E_L = 0 mV<br>E_U = 500 mV<br>5 Hz	E_L = 0 mV<br>E_U = 500 mV<br>25 Hz	E_L = 0 mV<br>E_U = 500 mV<br>100 Hz

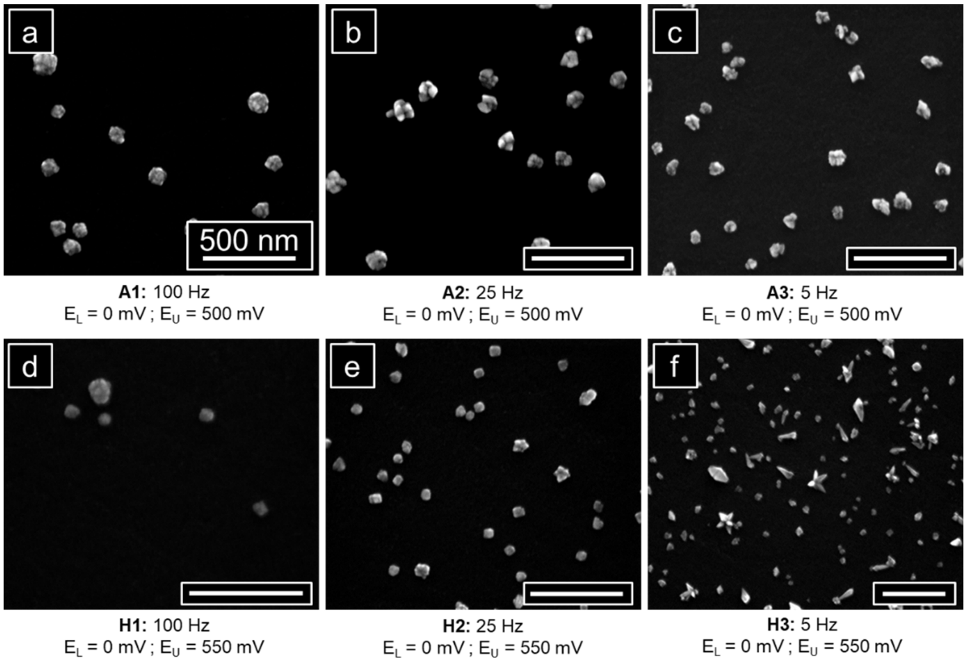

Figure S2. SEM images of Pd nanoparticles synthesized from a solution of 0.2 mM H_2PdCl_4 in 0.1 M $HClO_4$ via square wave deposition at frequencies of 5, 25, or 100 Hz. (All potentials vs. Ag/AgO QRCE; scale bars: 500 nm.)

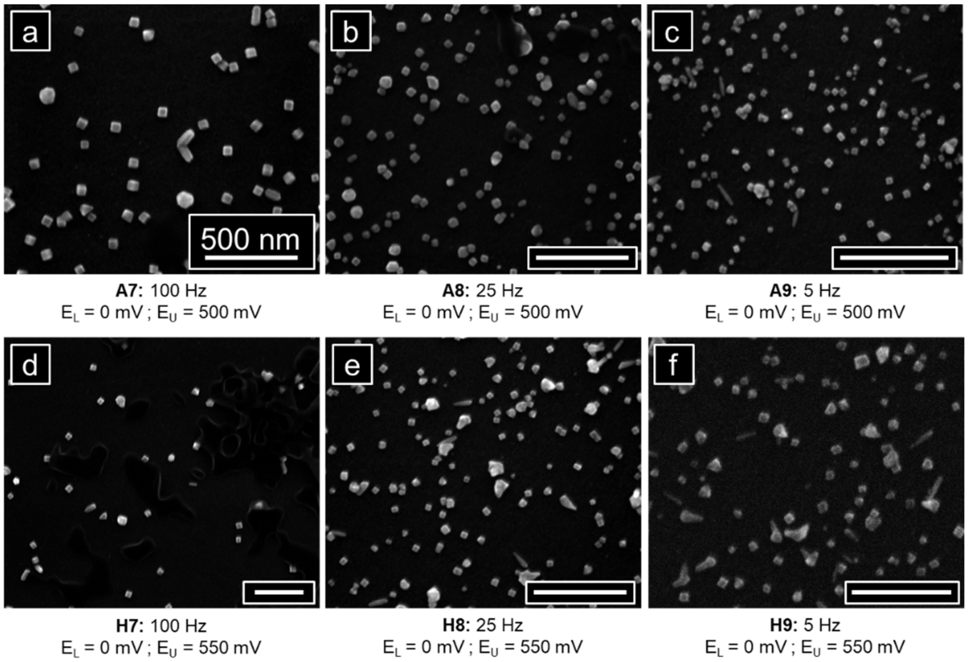

Figure S3. SEM images of Pd nanoparticles synthesized from a solution of 0.5 mM H_2PdCl_4 in 0.1 M $CTAHSO_4$ via square wave deposition at frequencies of 5, 25, or 100 Hz. (All potentials vs. Ag/AgO QRCE; scale bars: 500 nm.)

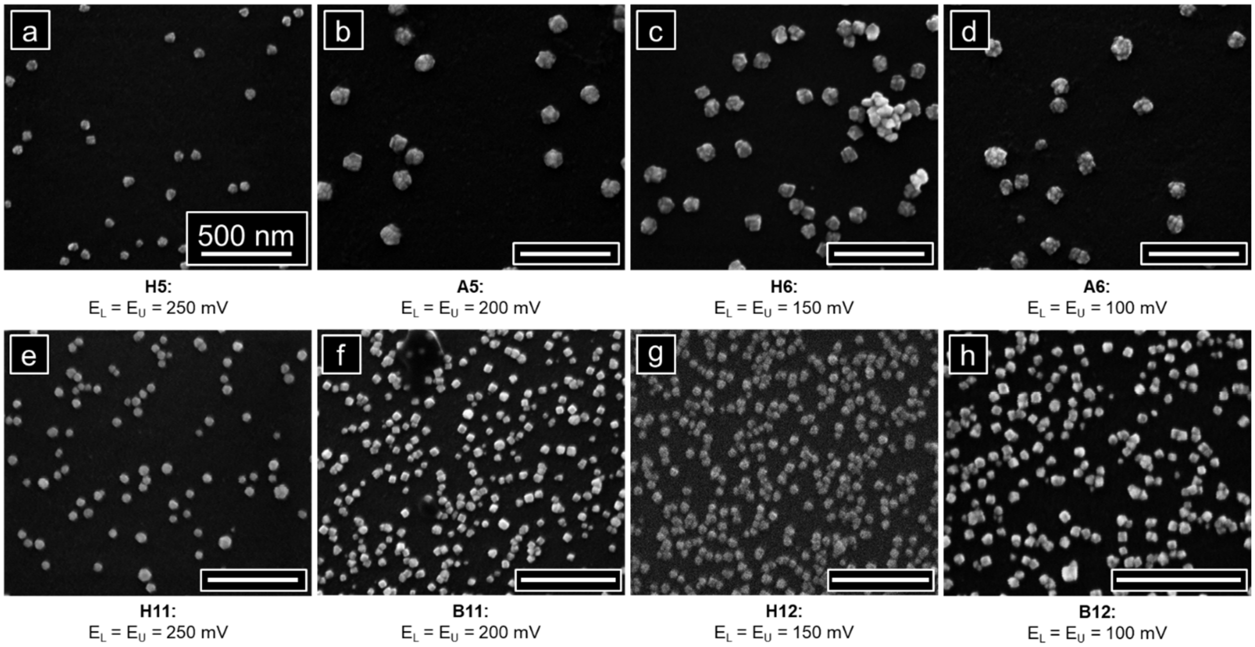

Figure S4. SEM images of Pd nanoparticles synthesized via constant potential deposition at varying potentials from a solution of (a-d) 0.2 mM H_2PdCl_4 in 0.1 M $HClO_4$ and (e-h) 0.5 mM H_2PdCl_4 in 0.1 M $CTAHSO_4$. (All potentials vs. Ag/AgO QRCE; scale bars: 500 nm.)

Table 2. Conditions of Screening Array for Optimization of Pd Cube Electrodeposition in 0.1 M $CTAHSO_4$

	12	11	10	9	8	7	6	5	4	3	2	1
	For all, unless otherwise specified:<br>E_{equil} = 500 mV for 100 ms; E_{nuc} = -200 mV for 100 ms<br>Frequency = 100 Hz; Deposition Time = 30 min<br>0.5 mM H_2PdCl_4 in 100 mM $CTAHSO_4$; All potentials vs. Ag/AgO QRCE											
**H**	E_L = 0 mV<br>E_U = 500 mV<br>0.25 mM<br>H_2PdCl_4	E_L = 0 mV<br>E_U = 500 mV<br>1 mM H_2PdCl_4	E_L = 0 mV<br>E_U = 500 mV<br>12.5 mM<br>$CTAHSO_4$	E_L = 0 mV<br>E_U = 500 mV<br>25 mM<br>$CTAHSO_4$	E_L = 0 mV<br>E_U = 500 mV<br>50 mM<br>$CTAHSO_4$	E_{nuc} for 200 ms,<br>then 0 mV for 2s<br>(no equil. step)	$E_L = E_U$<br>= 0 mV	$E_L = E_U$<br>= 100 mV	E_L = -100 mV<br>E_U = 500 mV	E_L = 100 mV<br>E_U = 500 mV	E_L = 0 mV<br>E_U = 300 mV	E_L = 0 mV<br>E_U = 500 mV
**G**	E_L = 0 mV<br>E_U = 500 mV<br>0.25 mM<br>H_2PdCl_4	E_L = 0 mV<br>E_U = 500 mV<br>1 mM H_2PdCl_4	E_L = 0 mV<br>E_U = 500 mV<br>12.5 mM<br>$CTAHSO_4$	E_L = 0 mV<br>E_U = 500 mV<br>25 mM<br>$CTAHSO_4$	E_L = 0 mV<br>E_U = 500 mV<br>50 mM<br>$CTAHSO_4$	E_{nuc} for 200 ms,<br>then 0 mV for 2s<br>(no equil. step)	$E_L = E_U$<br>= 0 mV	$E_L = E_U$<br>= 100 mV	E_L = -100 mV<br>E_U = 500 mV	E_L = 100 mV<br>E_U = 500 mV	E_L = 0 mV<br>E_U = 300 mV	E_L = 0 mV<br>E_U = 500 mV
**F**	E_L = 0 mV<br>E_U = 500 mV<br>0.25 mM<br>H_2PdCl_4	E_L = 0 mV<br>E_U = 500 mV<br>1 mM H_2PdCl_4	E_L = 0 mV<br>E_U = 500 mV<br>12.5 mM<br>$CTAHSO_4$	E_L = 0 mV<br>E_U = 500 mV<br>25 mM<br>$CTAHSO_4$	E_L = 0 mV<br>E_U = 500 mV<br>50 mM<br>$CTAHSO_4$	E_{nuc} for 200 ms,<br>then 0 mV for 2s<br>(no equil. step)	$E_L = E_U$<br>= 0 mV	$E_L = E_U$<br>= 100 mV	E_L = -100 mV<br>E_U = 500 mV	E_L = 100 mV<br>E_U = 500 mV	E_L = 0 mV<br>E_U = 300 mV	E_L = 0 mV<br>E_U = 500 mV
**E**	E_L = 0 mV<br>E_U = 500 mV<br>0.25 mM<br>H_2PdCl_4	E_L = 0 mV<br>E_U = 500 mV<br>1 mM H_2PdCl_4	E_L = 0 mV<br>E_U = 500 mV<br>12.5 mM<br>$CTAHSO_4$	E_L = 0 mV<br>E_U = 500 mV<br>25 mM<br>$CTAHSO_4$	E_L = 0 mV<br>E_U = 500 mV<br>50 mM<br>$CTAHSO_4$	E_{nuc} for 200 ms,<br>then 0 mV for 2s<br>(no equil. step)	$E_L = E_U$<br>= 0 mV	$E_L = E_U$<br>= 100 mV	E_L = -100 mV<br>E_U = 500 mV	E_L = 100 mV<br>E_U = 500 mV	E_L = 0 mV<br>E_U = 300 mV	E_L = 0 mV<br>E_U = 500 mV
**D**	$E_L = E_U$<br>= 100 mV<br>0.25 mM<br>H_2PdCl_4	$E_L = E_U$<br>= 100 mV<br>1 mM H_2PdCl_4	$E_L = E_U$<br>= 100 mV<br>12.5 mM<br>$CTAHSO_4$	$E_L = E_U$<br>= 100 mV<br>25 mM<br>$CTAHSO_4$	$E_L = E_U$<br>= 100 mV<br>50 mM<br>$CTAHSO_4$	E_{nuc} for 200 ms,<br>then 100 mV for<br>2s<br>(no equil. step)	200 ms<br>E_{nuc} duration	$E_L = E_U$<br>= 150 mV	$E_L = E_U$<br>= 50 mV	E_L = -50 mV<br>E_U = 500 mV	E_L = 50 mV<br>E_U = 500 mV	E_L = 0 mV<br>E_U = 400 mV
**C**	$E_L = E_U$<br>= 100 mV<br>0.25 mM<br>H_2PdCl_4	$E_L = E_U$<br>= 100 mV<br>1 mM H_2PdCl_4	$E_L = E_U$<br>= 100 mV<br>12.5 mM<br>$CTAHSO_4$	$E_L = E_U$<br>= 100 mV<br>25 mM<br>$CTAHSO_4$	$E_L = E_U$<br>= 100 mV<br>50 mM<br>$CTAHSO_4$	E_{nuc} for 200 ms,<br>then 100 mV for<br>2s<br>(no equil. step)	200 ms<br>E_{nuc} duration	$E_L = E_U$<br>= 150 mV	$E_L = E_U$<br>= 50 mV	E_L = -50 mV<br>E_U = 500 mV	E_L = 50 mV<br>E_U = 500 mV	E_L = 0 mV<br>E_U = 400 mV
**B**	$E_L = E_U$<br>= 100 mV<br>0.25 mM<br>H_2PdCl_4	$E_L = E_U$<br>= 100 mV<br>1 mM H_2PdCl_4	$E_L = E_U$<br>= 100 mV<br>12.5 mM<br>$CTAHSO_4$	$E_L = E_U$<br>= 100 mV<br>25 mM<br>$CTAHSO_4$	$E_L = E_U$<br>= 100 mV<br>50 mM<br>$CTAHSO_4$	E_{nuc} for 200 ms,<br>then 100 mV for<br>2s<br>(no equil. step)	200 ms<br>E_{nuc} duration	$E_L = E_U$<br>= 150 mV	$E_L = E_U$<br>= 50 mV	E_L = -50 mV<br>E_U = 500 mV	E_L = 50 mV<br>E_U = 500 mV	E_L = 0 mV<br>E_U = 400 mV
**A**	$E_L = E_U$<br>= 100 mV<br>0.25 mM<br>H_2PdCl_4	$E_L = E_U$<br>= 100 mV<br>1 mM H_2PdCl_4	$E_L = E_U$<br>= 100 mV<br>12.5 mM<br>$CTAHSO_4$	$E_L = E_U$<br>= 100 mV<br>25 mM<br>$CTAHSO_4$	$E_L = E_U$<br>= 100 mV<br>50 mM<br>$CTAHSO_4$	E_{nuc} for 200 ms,<br>then 100 mV for<br>2s<br>(no equil. step)	200 ms<br>E_{nuc} duration	$E_L = E_U$<br>= 150 mV	$E_L = E_U$<br>= 50 mV	E_L = -50 mV<br>E_U = 500 mV	E_L = 50 mV<br>E_U = 500 mV	E_L = 0 mV<br>E_U = 400 mV

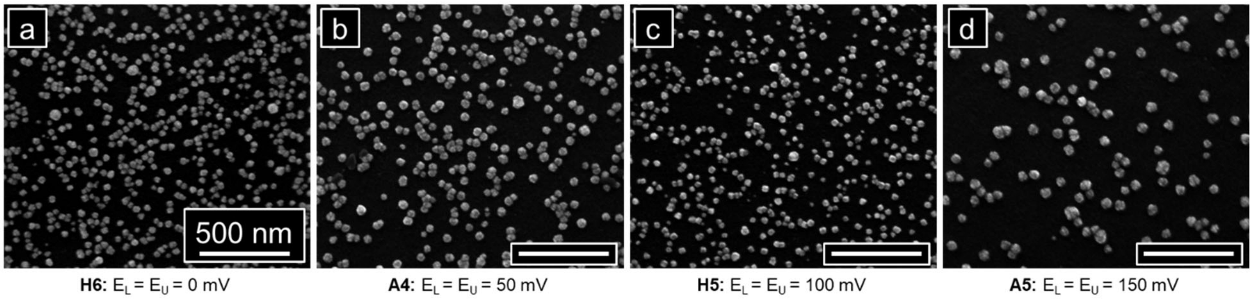

Figure S5. SEM images of Pd nanoparticles synthesized via constant potential deposition at varying potentials from a solution of 0.5 mM H_2PdCl_4 in 0.1 M $CTAHSO_4$. (All potentials vs. Ag/AgO QRCE; scale bars: 500 nm.)

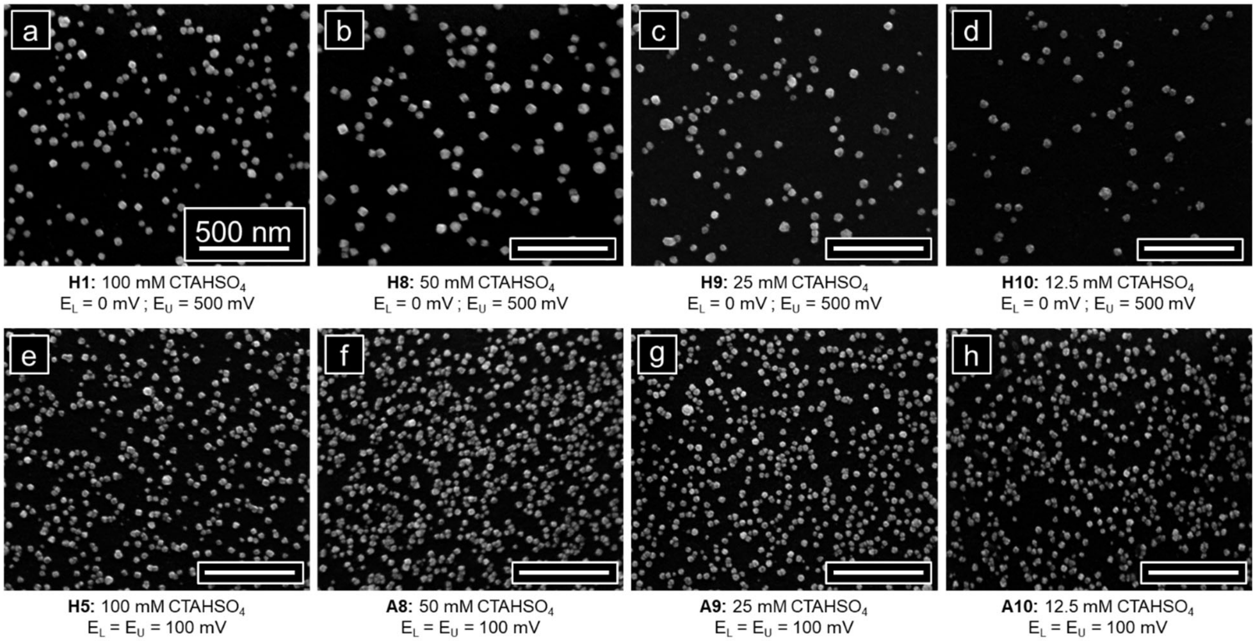

Figure S6. SEM images of Pd nanoparticles synthesized via (a-d) square wave deposition or (e-h) constant potential deposition from a solution of 0.5 mM H_2PdCl_4 in varying concentrations of $CTAHSO_4$. (All potentials vs. Ag/AgO QRCE; scale bars: 500 nm.)

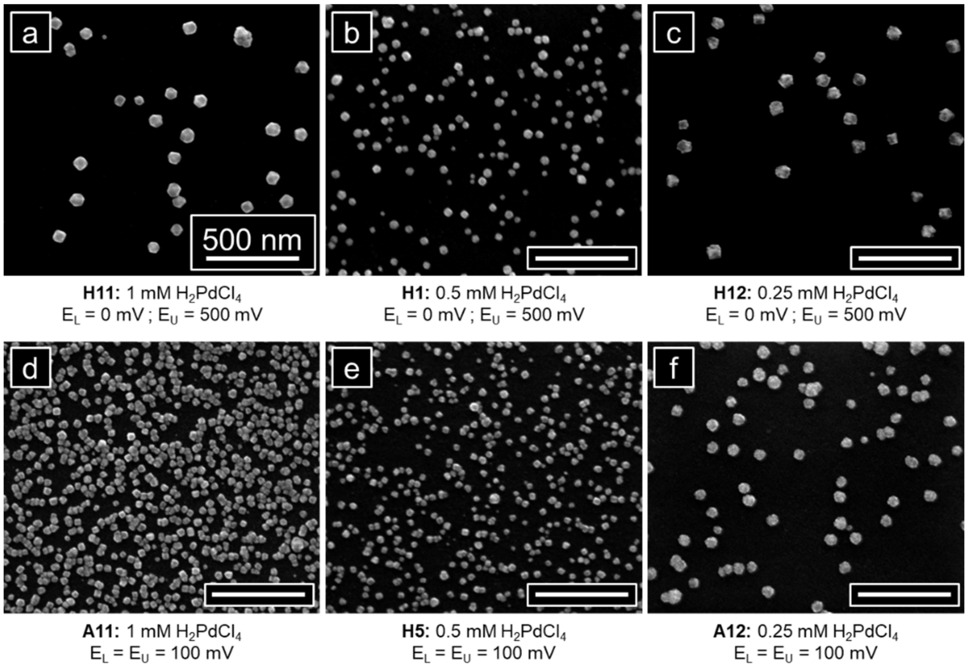

Figure S7. SEM images of Pd nanoparticles synthesized via (a-c) square wave deposition or (d-f) constant potential deposition from a solution of varying concentrations of H_2PdCl_4 in 0.1 M $CTAHSO_4$. (All potentials vs. Ag/AgO QRCE; scale bars: 500 nm.)

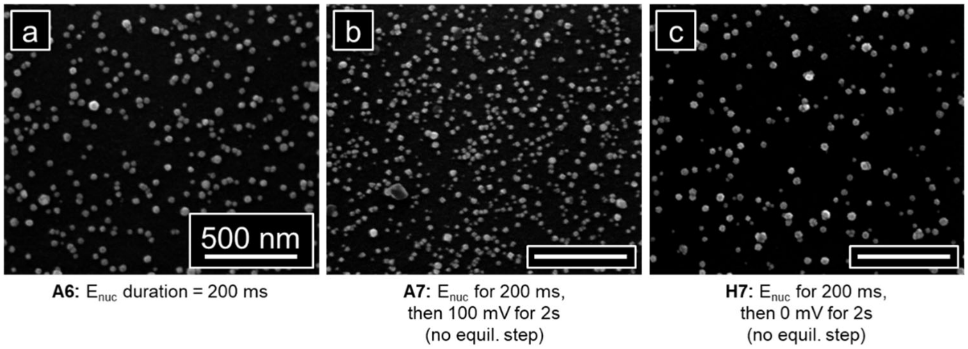

Figure S8. SEM images of Pd nanoparticles synthesized via square wave deposition (E_L = 0 mV; E_U = 500 mV) from a solution of 0.5 mM H_2PdCl_4 in 0.1 M $CTAHSO_4$, with modifications to the initial nucleation steps prior to the square wave. (All potentials vs. Ag/AgO QRCE; scale bars: 500 nm.)

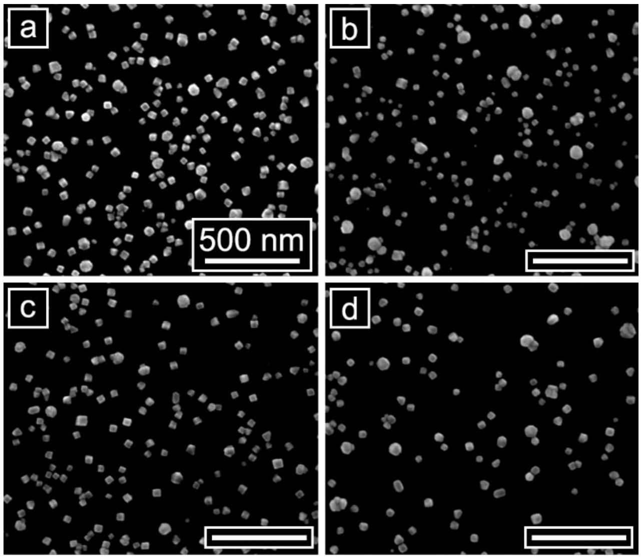

Figure S9. SEM images of Pd nanoparticles synthesized under the same electrodeposition conditions but in different wells (A3-D3 from the array in Table 2), showing that results are reproducible across different wells within an array. (Scale bars: 500 nm.)

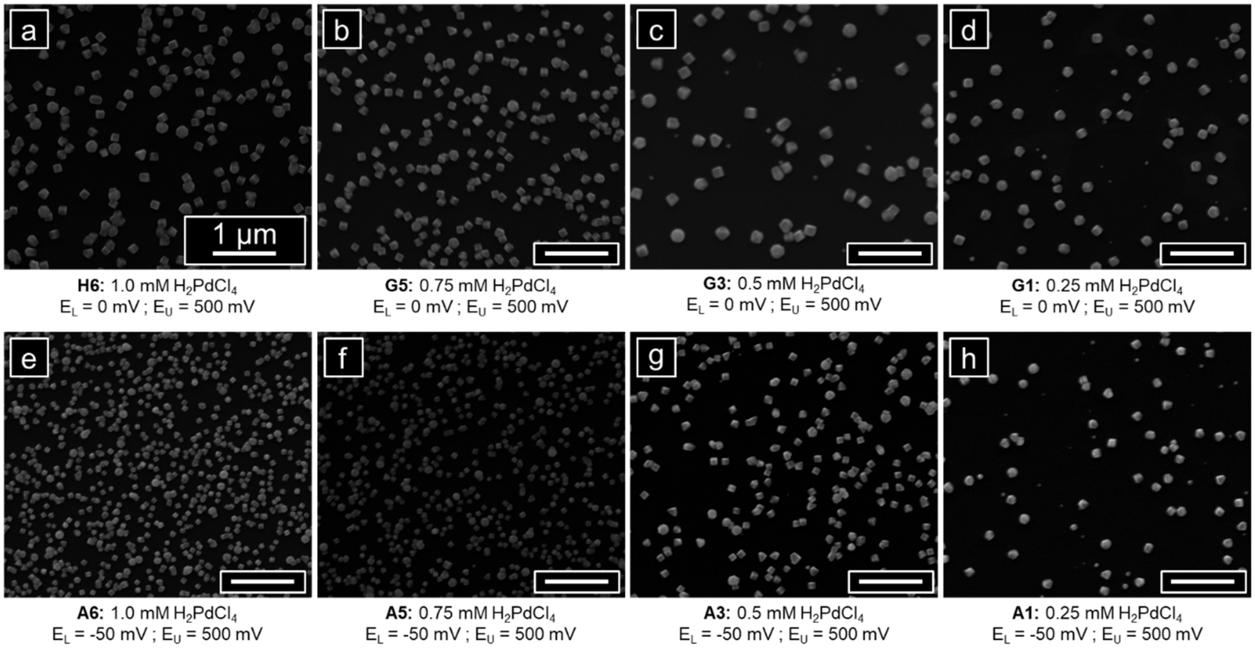

Figure S10. SEM images of Pd nanoparticles synthesized via square wave deposition for 60 min from a solution of varying concentrations of H_2PdCl_4 in 0.1 M $CTAHSO_4$. (All potentials vs. Ag/AgO QRCE; scale bars: 1 μm.)
